# A cross-sectional study of the spectrum of intraoperative findings in patients presenting with penetrating abdominal trauma at the Katutura and Windhoek Central State Hospitals, Windhoek, Namibia

**DOI:** 10.11604/pamj.2026.53.102.48337

**Published:** 2026-02-26

**Authors:** Anike Maritz, Pueya Mekondjo Nashidengo

**Affiliations:** 1 University of Namibia, Windhoek, Namibia,; 2 Surgery Department, Windhoek Central Hospital, University of Namibia, Windhoek, Namibia

**Keywords:** Abdominal injuries, wounds, penetrating, laparotomy, surgical procedures, operative, injury severity score, postoperative complications, Namibia

## Abstract

Penetrating abdominal trauma is associated with intraoperative findings. There is limited data describing patterns in the Namibian setting. This study aimed to describe the spectrum and frequency of intraoperative findings in patients undergoing operative management for penetrating abdominal trauma at Katutura and Windhoek Central State Hospitals in Windhoek, Namibia. A descriptive cross-sectional observational study was conducted among patients presenting with penetrating abdominal trauma who underwent operative management between April 2023 and February 2024. Data collected included sociodemographic characteristics, mechanism and anatomical distribution of injury, preoperative investigations, intraoperative findings, Injury Severity Score (ISS), Penetrating Abdominal Trauma Index (PATI), operative procedures, and short-term clinical outcomes. Thirty (30) patients were included, comprising 25 males (83.3%) and five females (16.7%), with a mean age of 31.6 years. Stab wounds accounted for 80% of injuries, followed by gunshot wounds (16.7%) and a fall from height (3.3%). Most patients sustained a single penetrating wound, with the epigastric region being the most frequently affected site (20%). The mean ISS was 10.6, and the mean PATI was 10.8. All patients underwent open laparotomy. The small bowel was the most commonly injured organ (36.67%). Four patients (13.3%) required intensive care unit admission, three required relaparotomy, and one in-hospital death occurred. The mean hospital length of stay was 10.63 days. In this cohort, penetrating abdominal trauma predominantly affected young males and was most commonly caused by stab wounds. The small bowel was the most frequently injured organ, and operative management was associated with low short-term mortality despite limited use of preoperative imaging.

## Introduction

Although penetrating abdominal trauma has been widely studied internationally [1-5], there is a paucity of published data describing the epidemiology and spectrum of intraoperative findings in the Namibian setting. In particular, limited information exists regarding the sociodemographic characteristics, mechanisms of injury, clinical management practices as outlined in national guidelines [6], and short-term outcomes within the Namibian healthcare context [7] of patients presenting with penetrating abdominal trauma. This study aimed to describe the spectrum of intraoperative findings in patients undergoing operative management for penetrating abdominal trauma at Katutura State Hospital and Windhoek Central State Hospital in Windhoek, Namibia.

The primary objective of this study was to describe the spectrum and frequency of intraoperative findings in patients undergoing operative management for penetrating abdominal trauma at Katutura State Hospital and Windhoek Central State Hospital in Windhoek, Namibia. Secondary objectives were to describe the sociodemographic characteristics of affected patients, the mechanisms and anatomical distribution of penetrating abdominal injuries, operative indications and injured organs, injury severity using the Injury Severity Score (ISS) and Penetrating Abdominal Trauma Index (PATI), and short-term clinical outcomes, including postoperative complications, need for relaparotomy, intensive care unit admission, length of hospital stay, and in-hospital mortality.

We asked the following research questions: what intraoperative injuries are most commonly encountered in patients undergoing laparotomy for penetrating abdominal trauma at the two state hospitals? What are the demographic and injury-related characteristics of these patients? And what are the short-term postoperative outcomes following operative management of penetrating abdominal trauma in this setting?

## Methods

**Research design and setting:** this was a quantitative descriptive cross-sectional observational study based on retrospective review of medical records, designed to address the predefined study objectives and research questions using descriptive and exploratory analyses. The study was conducted at Katutura State Hospital and Windhoek Central State Hospital, the only two state hospitals in Windhoek. Patients initially presented to Katutura State Hospital, with subsequent management occurring at either facility.

**Study population:** the study population included patients from all over Namibia who presented to Katutura Intermediate and Windhoek Central State hospitals who had penetrating abdominal injuries and had operative management.

**Sample and sampling method**

***Inclusion criteria:*** patients who present to Katutura Intermediate and Windhoek Central State Hospitals who have one or more penetrating abdominal trauma injuries, with indications for immediate or delayed operative intervention. Any subsequent laparotomy before the discharge of the non-operatively managed PAT cases is classified as delayed operative management (DOM). Patients of all ages will be included.

***Exclusion criteria:*** patients who sustained blunt abdominal trauma and non-abdominal traumatic injuries. The patient's medical and operative notes and laboratory and radiological records were reviewed to identify the cohort of PAT that was managed operatively.

**Data collection:** the target population was sampled after approval was obtained from the Ministry of Health and Social Services. The sample size was 30 patients. To avoid selection bias, the patients were recorded in order of presentation to the hospital. The data was collected by reading the patient details acquired from their medical records into an online password-protected electronic database on Google Forms. This researcher read and evaluated the medical records. The data capture form was an online password-protected Google Form. The information collected included demographic information (age, sex, address), the clinical course of the PAI (time of injury, time to admission, operative management indications), and intraoperative findings (liver injury, pancreatic injury, bowel injury, number of organ injuries). Only the researcher had access to the database linked to the Google Form. The data was collected by reviewing the patient's medical records. The database included fields such as basic patient demographics, presenting vital signs, mechanism of Penetrating Abdominal Trauma, number of penetrating traumatic insults, penetrating wound positions, presence of peritonism and evisceration, indication for laparotomy, radiological investigations and interventions, operative or non-operative management, laparotomy findings - therapeutic, non-therapeutic, or negative, abdominal visceral injuries and associated injuries.

**Data analysis:** EpiInfo was used for descriptive and exploratory statistical analysis. Descriptive analysis of the PAT patient included basic patient demographics; presenting vital signs; blood investigations; mechanism of PAT; number of penetrating traumatic insults; penetrating wound positions; presence of peritonism or visceral evisceration; indication for laparotomy; radiological investigations and interventions; operative or non-operative management; laparotomy findings - therapeutic, non-therapeutic, or negative; abdominal visceral injuries and associated injuries. Injury severity was described by the Injury Severity Score (ISS) and the Penetrating Abdominal Trauma Index (PATI). Outcome variables included Intensive Care Unit (ICU) admission, hospital length of stay (LOS), hospital readmission, relaparotomy, in-hospital complications, and mortality. Complications are any deviation from the usual postoperative course requiring pharmacological, endoscopic, interventional radiological, or surgical treatment. They were categorised according to the Clavien-Dindo classification system. For the descriptive analysis, continuous data were summarised using means and 95% confidence interval (95% CI) if normally distributed, medians and interquartile range (IQR) were used for non-normally distributed data. For inferential statistics, parametric tests such as Chi-square tests or, where appropriate, the non-parametric equivalent, were performed. A p-value of 0.05 was considered statistically significant.

**Ethical considerations:** ethical approval was obtained from the Ministry of Health and Social Services of Namibia through the University of Namibia Research Ethics Committee. As this was a retrospective record review, informed consent was waived. No patient identifiers were recorded, and data confidentiality was maintained throughout the study.

## Results

**Study population and sociodemographic characteristics:** during the study period from 7^th^ April 2023 to 19^th^ February 2024, a total of 30 patients who sustained penetrating abdominal trauma and underwent operative management met the inclusion criteria. These patients sustained a total of 34 penetrating abdominal wounds. The majority of patients were male (25/30; 83.3%), with five females (16.7%). The mean age was 31.6 years (SD 10.136; IQR 12). Most patients were unmarried (83.3%), while marital status was not documented for the remaining 16.7%. Educational and employment data were incompletely recorded: 80% of patients had no documented education level, 73.3% had no documented employment status, 20% were unemployed, and 6.7% were employed. Only one patient (3.3%) had a documented comorbidity (hypertension), while 96.7% had no recorded comorbidities. Most patients were transported to the hospital by ambulance (83.3%), with 10% arriving via private transport and 6.7% via public transport. Detailed sociodemographic characteristics are summarised in Table 1. The majority of injuries occurred in the Khomas region (56.7%), followed by Otjozondjupa (13.3%), Hardap (10%), and Omaheke (10%). The region of injury was not documented in 10% of cases. The geographic distribution of trauma incidents is shown in Table 2.

**Table 1:** socio-demographic characteristics and mechanisms of injury among patients undergoing operative management for penetrating abdominal trauma at Katutura State Hospital and Windhoek Central State Hospital, Windhoek, Namibia, April 2023-February 2024 (N = 30)

Sample size	30
Age (years mean ±SD)	31.6 ±10.1356
**Sex**	
Male	25 (83.33%)
Female	5 (16.67%)
**Marital status**	
Single	25 (83.3%)
Not recorded	5 (16.7%)
**Employment status**	
Employed	2 (6.7%)
Unemployed	6 (20%)
Not recorded	22 (73.3%)
**Type of injury**	
Stab wound	24 (80%)
Gunshot	5 (16.7%)
Fall from height	1 (3.3%)
Self-inflicted	1 (3.3%)

**Table 2:** regional distribution of trauma incidents among patients with penetrating abdominal injuries managed operatively at Katutura State Hospital and Windhoek Central State Hospital, Namibia, April 2023-February 2024 (N = 30)

Regions of Namibia	Frequency	Percent	Cumulative percent	Exact 95% lower confidence limit	Exact 95% upper confidence limit
Hardap	3	10.00%	10.00%	2.11%	26.53%
Khomas	17	56.67%	66.67%	37.43%	74.54%
Not recorded	3	10.00%	76.67%	2.11%	26.53%
Omaheke	3	10.00%	86.67%	2.11%	26.53%
Otjozondjupa	4	13.33%	100.00%	3.76%	30.72%
TOTAL	30	100.00%	100.00%		

**Mechanism, timing, and anatomical distribution of injuries:** the most common mechanism of injury was stab wounds, accounting for 24 patients (80%), including one suicidal stab attempt (3.3%). Gunshot wounds occurred in five patients (16.7%), and one patient (3.3%) sustained penetrating injury following a fall from height. Most patients (60%) sustained a single penetrating wound. Patients typically presented to the hospital on the day of the injury. A higher frequency of presentations occurred during the early morning hours, particularly between 04: 00 and 05: 00. Katutura suburb accounted for the highest number of cases (23.3%). The epigastric region was the most frequently affected anatomical site (20%), followed by the right and left iliac regions (13.33% each) and the right hypochondriac region (13.33%). Stab wounds were distributed across multiple abdominal regions, while gunshot wounds most commonly involved the right hypochondriac and right iliac regions. The suicidal stab attempt involved the epigastric region only. Stab wounds were distributed across multiple abdominal regions, most commonly involving the epigastric, right iliac, and right lumbar regions. Gunshot wounds most frequently involved the right hypochondriac and right iliac regions, while the single suicidal stab attempt involved the epigastric region only. The distribution of wound locations by mechanism of injury is shown in Table 3.

**Table 3:** distribution of penetrating abdominal wound locations by mechanism of injury among operatively managed patients at Katutura State Hospital and Windhoek Central State Hospital, Windhoek, Namibia, April 2023-February 2024 (N = 30)

	Fall from height	Gunshot	Stab	Stab, suicidal attempt	Total
Back	0	0	2	0	2
	0.00%	0.00%	8.70%	0.00%	6.67%
	0.00%	0.00%	100.00%	0.00%	100.00%
Epigastric region	0	1	4	1	6
	0.00%	20.00%	17.39%	100.00%	20.00%
	0.00%	16.67%	66.67%	16.67%	100.00%
Hypogastric region	0	0	1	0	1
	0.00%	0.00%	4.35%	0.00%	3.33%
	0.00%	0.00%	100.00%	0.00%	100.00%
Left hypochondriac region	1	0	2	0	3
	100.00%	0.00%	8.70%	0.00%	10.00%
	33.33%	0.00%	66.67%	0.00%	100.00%
Left iliac region	0	1	1	0	2
	0.00%	20.00%	4.35%	0.00%	6.67%
	0.00%	50.00%	50.00%	0.00%	100.00%
Left lumbar region	0	0	3	0	3
	0.00%	0.00%	13.04%	0.00%	10.00%
	0.00%	0.00%	100.00%	0.00%	100.00%
Right hypochondriac region	0	2	2	0	4
	0.00%	40.00%	8.70%	0.00%	13.33%
	0.00%	50.00%	50.00%	0.00%	100.00%
Right iliac region	0	0	4	0	4
	0.00%	0.00%	17.39%	0.00%	13.33%
	0.00%	0.00%	100.00%	0.00%	100.00%
Right lumbar region	0	1	1	0	2
	0.00%	20.00%	4.35%	0.00%	6.67%
	0.00%	50.00%	50.00%	0.00%	100.00%
Umbilical region	0	0	3	0	3
	0.00%	0.00%	13.04%	0.00%	10.00%
	0.00%	0.00%	100.00%	0.00%	100.00%
Total (incidence)	1	5	23	1	30
	100.00%	100.00%	100.00%	100.00%	100.00%
	3.33%	16.67%	76.67%	3.33%	100.00%

**Injury severity and operative indications:** the mean Injury Severity Score (ISS) was 10.6 (IQR 11). The organs associated with the highest individual injury severity scores were the liver (one case) and the small bowel (one case). The mean Penetrating Abdominal Trauma Index (PATI) was 10.8 (IQR 11). The most common indication for laparotomy was organ evisceration, occurring in nine patients, followed by hemodynamic instability in six patients. Preoperative imaging was limited to five patients (16.7%) who underwent computed tomography (CT), two patients underwent ultrasound examination, and two patients received both ultrasound and CT imaging before surgery.

**Intraoperative findings:** all 30 patients underwent open laparotomy. The small bowel was the most frequently injured organ, identified in 11 cases (36.67%), followed by the large bowel (22.22%). The most frequently encountered penetrating abdominal trauma index score was 4, occurring in 13.33% of patients, while an ISS of 9 was the most common injury severity score, accounting for 30% of cases. Associated extra-abdominal injuries were uncommon. The epigastric region was associated with the highest number of additional injuries, including one peripheral vascular injury and one upper thoracic injury. Overall, 24 patients had no additional injuries. No statistically significant association was observed between wound location and associated injuries (Chi-square = 41.35; p = 0.627).

**Postoperative outcomes and complications:** postoperatively, four patients (13.3%) required admission to the Intensive Care Unit, while the remaining 26 patients (86.7%) were managed in the general surgical wards. Relaparotomy was required in three patients. Postoperative complications, classified according to the Clavien-Dindo system, were most commonly Grade II (9 patients) or not applicable (9 patients). Five patients experienced Grade IV complications. There was one in-hospital death, resulting in a mortality rate of 3.3%. The mean hospital length of stay was 10.63 days, with a range from 1 to 34 days.

## Discussion

**Statement of principal findings:** this cross-sectional observational study described the spectrum of intraoperative findings and short-term outcomes in patients undergoing operative management for penetrating abdominal trauma at Katutura State Hospital and Windhoek Central State Hospital in Windhoek, Namibia. The cohort consisted predominantly of young males, with a mean age of 31.6 years, and stab wounds were the most common mechanism of injury. The epigastric region was the most frequently affected anatomical site, and the small bowel was the most commonly injured organ, accounting for 36.67% of cases. The mean Injury Severity Score was 10.6, and the mean Penetrating Abdominal Trauma Index was 10.8. Most patients were managed postoperatively in the general surgical wards, with a low rate of intensive care unit admission and a single in-hospital death.

**Comparison with existing literature:** the discrepancy in organ injury patterns compared to the South African study [4]-where the liver ranked highest, followed by the small bowel and colon—may be attributed to differences in weapon type, injury mechanisms, or sample size. The older mean age reported in the French study [5] (36 years versus 31.6 years in this study) suggests regional variability in the demographics of penetrating abdominal trauma. Moreover, while the Tanzanian study reported a male-to-female ratio of 5.5: 1 [3], this study did not calculate a ratio but demonstrated a strong male predominance (83.3%).

**Interpretation of findings:** the predominance of stab wounds in this cohort likely reflected local patterns of interpersonal violence in the urban Namibian setting. The high frequency of small bowel injuries may be explained by the anatomical location and mobility of the small intestine, which increase its susceptibility to penetrating mechanisms. The low rates of intensive care unit admission and in-hospital mortality observed may indicate timely operative intervention and appropriate patient selection for surgery. Despite limited access to preoperative imaging, operative decisions were primarily guided by clinical findings such as organ evisceration and hemodynamic instability. This approach is consistent with established trauma management principles that prioritise early laparotomy in patients with penetrating abdominal trauma who present with clear clinical indications, particularly in low-resource settings where advanced imaging may not be readily available [4,8].

**Clinical implications and future research:** the findings of this study provide baseline epidemiological and operative data that may assist clinicians and healthcare planners in understanding the burden and characteristics of penetrating abdominal trauma in a low-resource setting. Recognition of common injury mechanisms, anatomical patterns, and intraoperative findings may support improved surgical preparedness and resource allocation and may inform the refinement of local trauma management protocols in line with existing national guidelines [6].

Future research should focus on larger, multicentre studies with prospective data collection to better identify predictors of morbidity and mortality following penetrating abdominal trauma. Further evaluation of pre-hospital care systems, the role of diagnostic imaging, and long-term postoperative outcomes would enhance understanding of trauma care pathways and patient prognosis in Namibia and similar settings [1,3,5,7].

**Study strengths and limitations:** a key strength of this study was the uniform operative management of all included patients, allowing for direct and consistent assessment of intraoperative findings and providing granular data on injury patterns, operative indications, and outcomes in a low-resource setting where published data are limited. However, several limitations should be considered. The small sample size (n = 30) limited statistical power and the ability to identify predictors of morbidity and mortality, particularly given the low number of deaths recorded (3.3%). The cross-sectional design and reliance on retrospective medical record review resulted in incomplete sociodemographic data, including employment and education status. In addition, limited access to preoperative imaging, with only five patients undergoing CT scans, restricted conclusions regarding diagnostic utility. As the study was conducted in two state hospitals within a single urban area, the findings may not be generalisable to rural settings or other regions of Namibia. Despite these limitations, the study provides valuable baseline data on penetrating abdominal trauma in the Namibian context and highlights areas for future research and system improvement.

## Conclusion

This study found that penetrating abdominal trauma in Windhoek predominantly affects young males, with stab wounds being the most common mechanism of injury. The small bowel was the most frequently injured organ, and despite limited access to advanced diagnostic imaging, operative management was associated with favourable short-term outcomes and low in-hospital mortality. These findings contribute important descriptive data to the limited body of literature on penetrating abdominal trauma in Namibia and comparable low-resource environments.

### What is known about this topic

- Penetrating abdominal trauma, commonly caused by stab wounds and gunshot injuries, is a major contributor to trauma-related morbidity and mortality, particularly among young adults; injury severity is influenced by factors such as mechanism of injury, projectile characteristics, and the organs involved;
- Globally, penetrating abdominal trauma predominantly affects males, with stab wounds being the most common mechanism and the small bowel is frequently injured; outcomes vary widely depending on injury severity, timeliness of surgical intervention, and the presence of associated injuries;
- Timely clinical decision-making remains central to the management of penetrating abdominal trauma, with laparotomy indicated in patients presenting with hemodynamic instability, peritonitis, or signs of intra-abdominal injury. In low-resource settings, limited access to advanced diagnostic imaging continues to influence management strategies.

### What this study adds

- This study provides the first Namibian description of operatively managed penetrating abdominal trauma, showing that cases predominantly involved young males (mean age 31.6 years), were most commonly caused by stab wounds (80%), and most frequently involved the epigastric region, with the small bowel being the most commonly injured organ (36.67%);
- The study documents moderate injury severity in this cohort (mean ISS 10.6; mean PATI 10.8), with universal open laparotomy, low ICU admission (13.3%), and low in-hospital mortality (3.3%) despite limited use of preoperative imaging.
